# Supplementary material for: Study of pulsed electrostatic field (PESF) in the perfusion of peripheral tissues: Microangiopathy, nutrition and quality of perceiver life
Source: PLoS One. 2022 Sep 15;17(9):e0268497. doi: 10.1371/journal.pone.0268497 (PMC9477350; doi:10.1371/journal.pone.0268497)
Supplement: Attachment — Submitted filename: Response to Reviewers.docx [file pone.0268497.s001.docx]

Dear Reviewers,

thanks for constructive comments which made it possible to improve the paper.

1. The entire manuscript needs to be checked for grammar and composition. There are significant errors in sentence structure.

We have revised grammar composition

2. Are the authors following a standard guideline for clinical research studies - like STROBE - since this appears to be observational? This is unclear.

Our study is an observational and retrospective clinical report using the electronic archive present in our Operating Unit in according of STROBE criteria

3. Authors make a lot of assumptions based on very little data that is not clearly laid out. Details below:
a. Figure 1 - very hard to follow - the authors use the words no change (gp B) and homogeneity (gp A) - what is the difference? The figure shows 5 components in Gp B panel and 6 components in Gp A panel. Also note - the word used by authors is componenti - which needs to be corrected. The authors cannot expect a viewer of that figure to know at-a-glance what the difference is pre and post treatment in groups A and B. There must be a clearer representation of the exact differences pointed out or shown in a different format.

We have clarified Figure 1 and correct word mistake.

b. Authors describe measuring azotaemia levels - but where is the figure to show actual numbers? Only statistics are descriptively included.

We have insert azotaemia numbers

b. Although the authors are comparing two cohorts that are age-matched and matched for gender - there is insufficient demographic information to be clear about how other underlying conditions could impact the outcomes of their studies - not discussed at all.

The two groups were uniform and showed no noteworthy statistical differences

c. It is unclear how soon after the treatment the questionnaire was applied - so is the effect in group A short term or long term?

The effects were evaluated just after the treatment, and it would be interesting to re-evaluate them in the long term

d. Insufficient details in methodology: What were the simulated and actual test parameters? The authors reference a paper but there needs to be a brief summary of what was applied in this study.

We have implemented the methods even though there are references from our previous studies where the treatment details are present

e. The authors point out that NO levels are increased in Group A after treatment. Yet they do not discuss what the significance of this might be? Are the levels physiologically relevant to make an impact?

f. In all other data tables - the pre treatment levels were comparable between group A and B. However in table 3 - group B pretreatment seems quite high (equation (ln) column) compared to the same cohort in group A - can the authors explain that difference?

The pre of group B is not significantly different from group A

g. The discussion section is very wordy and reads like a general review - instead of specific and focused comments based on the actual observations they made.

Thanks we have remodeled the discussion

Reviewer #2: Liani et al., studied the effect of of pulsed electrostatic field (PESF) in the perfusion of peripheral tissues: microangiopathy, nutrition and quality of perceiver life. The manuscript has raised some major concerns.

1. In Table 2 the difference in values in pre-and post of group A is not substantial (though the p value is less than 0.05). The SD is large and the number of samples is not that big. Same type of differences are seen in Group B but not significant. The authors must show individual data pints in the form of graph and confirm the p values.

We confirm the statistics reported

2. The title claims nutrition which is not at all mentioned in the results or discussion

We have insert nutrition profile in discussion

3. Figure 1 is not clear.

We have clarified Figure 1 and correct word mistake.

Reviewer #3: The study is interesting, was rigorously done for 5 years, the perfusion of peripheral tissues employing employing pulsed electrostatic field was novel, however they were significant gaps, that authors could address these following gaps

1. There are literature evidences suggesting that there is increase in nitric oxide levels & sensitivity with weight loss, authors in the table 2 talk about significant difference in weight loss (p = 0.007), the control group had no weight loss, there is no discussion how the weight loss impacts (given in the table 2), the nitric oxide levels in case of PESF treated and untreated groups, Authors could include few lines in discussion if there is any positive co-relation between weight loss, PESF, and Nitric oxide levels with appropriate references.

We have introduced this aspect into the discussion

2. There is no clear elaboration on why there are no molecular markers included in the study for determining the microangiopathy recovery or progression. Authors could possible include one blood marker to indicate is due recovery due to synergistic effect of PESF other than NO levels.

Thanks for the tip we will keep it in consideration for future studies

3. The study would have been more interesting, if the authors could include any other treatment of microangiopathy (example but not limited) total triterpenic fraction of Centella asiatica (TTFCA) as another control group to determine the how positive is PESF bringing the life changes in microangiopathy

We excluded patients with major arteries occlusion, valued with doppler test and patients who used: polyphenols, antioxidants for their effects on the cellular signal; phytotherapics (eg. Centella asiatica extracts) for their known effects on endothelial cells; anabolic drugs for their effects on metabolism; and psychotropic drugs. we believe it is right to specify that we are aware of the effects of biofavonoids and phytotherapeutic substances including the Total Triterpenic Fraction (FTTCA) of Centella asiatica. Both categories of products act on the endothelium and reduce edema. We have previously dealt with arterial ulcers[20]. The derivatives of Centella Asiatica, applied locally, favour the healing of venous ulcers. Centella asiatica improves the supply of O2 to tissues.
