# Supplementary material for: Study of pulsed electrostatic field (PESF) in the perfusion of peripheral tissues: Microangiopathy, nutrition and quality of perceiver life
Source: PLoS One. 2022 Sep 15;17(9):e0268497. doi: 10.1371/journal.pone.0268497 (PMC9477350; doi:10.1371/journal.pone.0268497)
Supplement: Attachment — Submitted filename: Response to Reviewers_2.docx [file pone.0268497.s002.docx]

| **Date:** | Dec 06 2021 07:59AM |
| --- | --- |
| **To:** | "Rossella Liani" rossellaliani@yahoo.it |
| **From:** | "PLOS ONE" plosone@plos.org |
| **Subject:** | PLOS ONE Decision: Revision required [PONE-D-21-05955R1] |

PONE-D-21-05955R1
Study of pulsed electrostatic field (PESF) in the perfusion of peripheral tissues: microangiopathy, nutrition and quality of perceiver life.
PLOS ONE

Additional Editor Comments:

Although reviewers have acknowledge the significant efforts of authors to revise this manuscript, they have recommended some more changes in order to make this study robust for publication. Please include individual data points for conclusions drawn for Table 2. Reviewer 3 comments are mentioned below:

Reviewer 3:

1. There are literature evidences suggesting that there is increase in nitric oxide levels
& sensitivity with weight loss, authors in the table 2 talk about significant difference in
weight loss (p = 0.007), the control group had no weight loss, there is no discussion
how the weight loss impacts (given in the table 2), the nitric oxide levels in case of
PESF treated and untreated groups, Authors could include few lines in discussion if
there is any positive co-relation between weight loss, PESF, and Nitric oxide levels with
appropriate references.

Response: We have introduced this aspect into the discussion

Reviewer 3: Could you please elaborate the discussion further about positive co-relation between weight loss, PESF, and Nitric oxide levels with appropriate references.

Response: We have introduced this aspect into the discussion

2. There is no clear elaboration on why there are no molecular markers included in the
study for determining the microangiopathy recovery or progression. Authors could
possible include one blood marker to indicate is due recovery due to synergistic effect
of PESF other than NO levels.

Response: Thanks for the tip we will keep it in consideration for future studies

Reviewer 3: Could you please add a section on how circulating endothelial cells (CECs) are a reliable marker of disease activity in a variety of vascular disorders. If possible an experiment to determine the increase in count of endothelial cell post PESF.

Response: We have introduced this aspect into the discussion but the study is over and the CEC are valued on fresh sample (DOI: 10.1016/j.jim.2012.03.007; DOI: 10.1038/s41598-021-88941-x)

3. The study would have been more interesting, if the authors could include any other
treatment of microangiopathy (example but not limited) total triterpenic fraction of
Centella asiatica (TTFCA) as another control group to determine the how positive is
PESF bringing the life changes in microangiopathy

Response: We excluded patients with major arteries occlusion, valued with doppler test and
patients who used: polyphenols, antioxidants for their effects on the cellular signal;
phytotherapics (eg. Centella asiatica extracts) for their known effects on endothelial
cells; anabolic drugs for their effects on metabolism; and psychotropic drugs. we
believe it is right to specify that we are aware of the effects of biofavonoids and
phytotherapeutic substances including the Total Triterpenic Fraction (FTTCA) of
Centella asiatica. Both categories of products act on the endothelium and reduce
edema. We have previously dealt with arterial ulcers[20]. The derivatives of Centella
Asiatica, applied locally, favour the healing of venous ulcers. Centella asiatica
improves the supply of O2 to tissues.

Reviewer 3: Could you please include a patient group subjected through a different mode of treatment for microangiopathy as a positive control group

Response: Thanks for the tip we will keep it in consideration for future studies, but the study is over


Reviewer's Responses to Questions

**Comments to the Author**

1. If the authors have adequately addressed your comments raised in a previous round of review and you feel that this manuscript is now acceptable for publication, you may indicate that here to bypass the “Comments to the Author” section, enter your conflict of interest statement in the “Confidential to Editor” section, and submit your "Accept" recommendation.

Reviewer #1: (No Response)

Reviewer #2: (No Response)

Reviewer #3: (No Response)

2. Is the manuscript technically sound, and do the data support the conclusions?

The manuscript must describe a technically sound piece of scientific research with data that supports the conclusions. Experiments must have been conducted rigorously, with appropriate controls, replication, and sample sizes. The conclusions must be drawn appropriately based on the data presented.

Reviewer #1: Partly

Reviewer #2: Yes

Reviewer #3: (No Response)

3. Has the statistical analysis been performed appropriately and rigorously?

Reviewer #1: Yes

Reviewer #2: No

Reviewer #3: (No Response)

4. Have the authors made all data underlying the findings in their manuscript fully available?

The [PLOS Data policy](http://www.plosone.org/static/policies.action#sharing) requires authors to make all data underlying the findings described in their manuscript fully available without restriction, with rare exception (please refer to the Data Availability Statement in the manuscript PDF file). The data should be provided as part of the manuscript or its supporting information, or deposited to a public repository. For example, in addition to summary statistics, the data points behind means, medians and variance measures should be available. If there are restrictions on publicly sharing data—e.g. participant privacy or use of data from a third party—those must be specified.

Reviewer #1: (No Response)

Reviewer #2: Yes

Reviewer #3: (No Response)

5. Is the manuscript presented in an intelligible fashion and written in standard English?

PLOS ONE does not copyedit accepted manuscripts, so the language in submitted articles must be clear, correct, and unambiguous. Any typographical or grammatical errors should be corrected at revision, so please note any specific errors here.

Reviewer #1: (No Response)

Reviewer #2: Yes

Reviewer #3: (No Response)

6. Review Comments to the Author

Please use the space provided to explain your answers to the questions above. You may also include additional comments for the author, including concerns about dual publication, research ethics, or publication ethics. (Please upload your review as an attachment if it exceeds 20,000 characters)

Reviewer #1: The reviewers have made a good effort to improve their manuscript. Few points that need addressing in order to accept

1. While the authors did clean up most of their grammatical issues, there are still some that need revising. If you are able to get a professional english editing service to help, that might be very useful. Examples:
a. Patients treated with PESF were improved circulating nitric oxide levels - should read patients treated with PESF 'showed' improved...
b. improper and incomplete internal editing: In elders with T2DM and chronic kidney disease (CKD), without
macrovascular precedents, microangiopathy effects the early atrophy of the brain matter of the frontal
and temporal lobes and it affects memory, the executive functions and ultimately the behaviourFare
clic o toccare qui per immettere il testo.[12].
c. present in our Operating Unit in according of STROBE criteria - should read - present in our operating unit in "accordance with or following" STROBE criteria.

Thanks for the suggestions, we have corrected in the text

2. No response given to: e. The authors point out that NO levels are increased in Group A after treatment. Yet
they do not discuss what the significance of this might be? Are the levels
physiologically relevant to make an impact?

Perhaps the authors did make edits to account for this but it is not immediately clear.

Response: We have improved this aspect into the discussion

3. I don't see figure legends included for the 2 figures.

Response: Thanks. We have included figure legends for the 2 figures

4. Table cannot be shortened to Tab. Please write out the word Table.

Response: Thanks.

I think with these edits, the manuscript may be ok to be accepted for publication.

Reviewer #2: In Table 2 the difference in values in pre-and post of group A is not substantial
(though the p value is less than 0.05). The SD is large and the number of samples is
not that big. Same type of differences are seen in Group B but not significant. The
authors MUST SHOW individual data pints in the form of graph and confirm the p values.

Response: Thanks, but the p value is at most equal to 0.021 in Table 2, and not near to 0.05, full significance P value

Reviewer #3: (No Response)

7. PLOS authors have the option to publish the peer review history of their article ([what does this mean?](https://journals.plos.org/plosone/s/editorial-and-peer-review-process#loc-peer-review-history)). If published, this will include your full peer review and any attached files.


If you choose “no”, your identity will remain anonymous but your review may still be made public.


**Do you want your identity to be public for this peer review?** For information about this choice, including consent withdrawal, please see our [Privacy Policy](https://www.plos.org/privacy-policy).

Reviewer #1: No

Reviewer #2: No

Reviewer #3: No
